# Plasma Concentrations of Lysophosphatidic Acid and Autotaxin in Abstinent Patients with Alcohol Use Disorder and Comorbid Liver Disease

**DOI:** 10.3390/biomedicines9091207

**Published:** 2021-09-13

**Authors:** María Flores-López, Nuria García-Marchena, Francisco Javier Pavon, Estrella Lara, Oscar Porras-Perales, Pedro Araos, Nerea Requena-Ocaña, Sandra Torres-Galván, M. Carmen Mañas-Padilla, Gabriel Rubio, Juan Suárez, Luis J. Santín, Fernando Rodríguez de Fonseca, Estela Castilla-Ortega, María I. García-Fernández, Antonia Serrano

**Affiliations:** 1 Instituto de Investigación Biomédica de Málaga—IBIMA, 29071 Málaga, Spain; maria.flores@ibima.eu (M.F.-L.); nuria.garcia@ibima.eu (N.G.-M.); javier.pavon@ibima.eu (F.J.P.); oscar.porras@ibima.eu (O.P.-P.); paraos@uma.es (P.A.); nerea.requena@ibima.eu (N.R.-O.); sandra.torres2594@gmail.com (S.T.-G.); juan.suarez@uma.es (J.S.); luis@uma.es (L.J.S.); fernando.rodriguez@ibima.eu (F.R.d.F.); 2 Unidad de Gestión Clínica de Salud Mental, Hospital Regional Universitario de Málaga, 29010 Málaga, Spain; 3 Departamento de Psicobiología y Metodología de las Ciencias del Comportamiento, Facultad de Psicología, Universidad de Málaga, 29071 Málaga, Spain; mmp241@uma.es; 4 Unidad de Adicciones-Servicio de Medicina Interna, Institut d’Investigació en Ciències de la Salut Germans Trias i Pujol (IGTP), 08916 Badalona, Spain; 5 Unidad de Gestión Clínica del Corazón, Hospital Universitario Virgen de la Victoria de Málaga, 29010 Málaga, Spain; 6 Centro de Investigación Biomédica en Red de Enfermedades Cardiovasculares (CIBERCV), Instituto de Salud Carlos III, 28029 Madrid, Spain; 7 Departamento de Fisiología Humana, Histología Humana, Anatomía Patológica Y Educación Física y Deportiva, Facultad de Medicina, Universidad de Málaga, 29071 Málaga, Spain; elara@uma.es; 8 Facultad de Farmacia, Universidad Complutense de Madrid, 28040 Madrid, Spain; 9 Servicio de Psiquiatría, Hospital Universitario 12 de Octubre, 28041 Madrid, Spain; gabriel.rubio@salud.madrid.org; 10 Departamento Anatomía Humana, Medicina Legal e Historia de la Ciencia, Facultad de Medicina, Universidad de Málaga, 29071 Málaga, Spain

**Keywords:** lysophosphatidic acid, autotaxin, alcohol use disorder, liver disease, comorbidity

## Abstract

Lysophosphatidic acid (LPA) is an endogenous lysophospholipid and a bioactive lipid that is synthesized by the enzyme autotaxin (ATX). The ATX–LPA axis has been associated with cognitive dysfunction and inflammatory diseases, mainly in a range of nonalcoholic liver diseases. Recently, preclinical and clinical evidence has suggested a role of LPA signaling in alcohol use disorder (AUD) and AUD-related cognitive function. However, the ATX–LPA axis has not been sufficiently investigated in alcoholic liver diseases. An exploratory study was conducted in 136 participants, 66 abstinent patients with AUD seeking treatment for alcohol (alcohol group), and 70 healthy control subjects (control group). The alcohol group was divided according to the presence of comorbid liver diseases (i.e., fatty liver/steatosis, alcoholic steatohepatitis, or cirrhosis). All participants were clinically evaluated, and plasma concentrations of total LPA and ATX were measured using enzyme-linked immunosorbent assays. Data were primarily analyzed using analysis of covariance (ANCOVA) while controlling for age, body mass index, and sex. Logistic regression models were created to assess the association of the ATX–LPA axis and AUD or liver disease. LPA and ATX were log_10_-transformed to fit the assumptions of parametric testing.The main results were as follows: total LPA and ATX concentrations were dysregulated in the alcohol group, and patients with AUD had significantly lower LPA (F_(1,131)_ = 10.677, *p* = 0.001) and higher ATX (F_(1,131)_ = 8.327, *p* = 0.005) concentrations than control subjects; patients with AUD and liver disease had significantly higher ATX concentrations (post hoc test, *p* < 0.05) than patients with AUD but not liver disease; significant correlations between AUD-related variables and concentrations of LPA and ATX were only found in the non-liver disease subgroup (the duration of alcohol abstinence with LPA and ATX (r = +0.33, *p* < 0.05); and the severity of AUD with ATX (rho = −0.33, *p* < 0.05)); and a logistic regression model with LPA, ATX, and AUD-related variables showed an excellent discriminative power (area under the curve (AUC) = 0.915, *p* < 0.001) for distinguishing patients with AUD and comorbid liver disease. In conclusion, our data show that the ATX–LPA axis is dysregulated in AUD and suggest this lipid signaling, in combination with relevant AUD-related variables, as a reliable biomarker of alcoholic liver diseases.

## 1. Introduction

Alcohol is the most commonly used drug in the general population worldwide. Although many individuals regularly drink alcohol in moderate doses with no significant adverse consequence, a substantial number develop alcohol use disorders (AUDs), which represents a major health problem with a devasting impact on individuals and society. In addition to AUD, chronic alcohol consumption is associated with a higher risk of suffering other psychiatric disorders (e.g., mood disorders, anxiety, and psychotic disorders) and organic diseases (e.g., gastrointestinal and accessory digestive organ diseases) [1,2,3,4]. It is, therefore, crucial to identify potential biomarkers for AUD and comorbid diseases in these patients, which can be relevant to develop new strategies for their stratification and treatment.

Among potential biomarkers, recent studies have linked the lysophosphatidic acid (LPA, 1- or 2-acyl-sn-glycerol 3-phosphate) species to alcohol-related behaviors and AUD [5,6,7]. LPA is an endogenous bioactive lipid with intercellular signaling properties that is detected in various biological samples, such as saliva, activated platelets, semen, or cerebrospinal fluid, though plasma is the major source of this lipid mediator [8]. Autotaxin (ATX) is the major enzyme responsible for LPA production and maintaining LPA concentration in blood, which is an extracellular lysophospholipase D (LPD) secreted by a variety of cells and tissues [9,10]. In the metabolic pathway of LPA, phospholipases A1 and A2 (PLA_1/2_) convert membrane phospholipids into lysophospholipids; these membrane-derived lipids are substrates for ATX to produce distinct LPA species [9,10,11]. In addition, PLA_1/2_ isozymes can also directly produce circulating LPA to a lesser extent than ATX from phosphatidic acid (PA) [10].

The biological actions of LPA are mediated by a complex family of G-protein-coupled membrane receptors (LPA_1-6_) that are ubiquitously distributed across the body tissues [12,13]. Acting mainly through the LPA_1_ receptor, LPA plays a role as a key mediator of physiological, developmental, and pathophysiological processes by enhancing cellular functions such as proliferation, differentiation, motility, and survival [9,12]. In this way, exogenous central LPA administration in rodents stimulates neuroplasticity, including adult hippocampal neurogenesis, and potentiates cognitive function at acute and repeated doses [14,15]. However, both LPA and ATX have also been implicated in the pathogenesis of many diseases and chronic inflammatory conditions such as atherosclerosis, fibrosis, neurodegenerative illnesses, and cancer [16]. Thus, plasma concentrations of LPA and ATX might be used as potential biomarkers for the diagnosis and severity of relevant diseases, and modulators of the ATX–LPA axis emerge as potential therapeutic targets [10,17].

In earlier preclinical studies from our group, we have suggested the role of LPA and its LPA_1_ receptor in alcohol-related behaviors. We have shown that mice lacking the LPA_1_ receptor (LPA_1_-null mice) display a notably increased voluntary alcohol drinking and more tolerance to the sedative effects of alcohol compared to their control counterparts [6]. Although these results may be attributed to neurodevelopmental alterations in the LPA_1_-null mice, alcohol-related behaviors are also modulated by systemic administration of the LPA_1/3_ receptor antagonist ki16425 in non-transgenic rodents [6,7]. Moreover, while the plasma levels of LPA are increased in alcohol-withdrawn mice relative to drug-naïve controls, the plasma levels of ATX are decreased [7]. In addition to these preclinical observations, we have recently examined LPA concentrations in the plasma of abstinent patients diagnosed with AUD [5]. This exploratory study revealed that plasma concentrations of LPA in patients with AUD are downregulated but correlate with plasma concentrations of relevant different growth factors (i.e., brain-derived neurotrophic factor (BDNF) and insulin-like growth factor-1 (IGF-1)). In addition, the decreased concentrations of LPA were found to be associated with cognitive impairments, which suggests that LPA might be a reliable marker for the detection of executive dysfunction linked to pathological alcohol use [5]. Although the dysregulation of LPA and cognitive impairment in AUD have been explored considering the high prevalence of psychiatric comorbidity, the potential association with other common non-psychiatric diseases (e.g., alcoholic liver diseases) has not been sufficiently examined.

In this regard, accessory digestive organ diseases are common in patients with AUD and have been associated with cognitive dysfunction, mainly in a range of liver diseases. Moreover, several clinical studies have suggested a potential role for ATX as a biomarker of nonalcoholic fatty liver disease (NAFLD) and steatohepatitis (NASH) [18,19,20,21].

Consequently, we hypothesize that patients with a history of AUD have differences in the ATX–LPA axis in comparison with healthy controls, and these alterations could be associated with the presence of liver diseases. Thus, the main aim of this study was to determine the plasma concentrations of LPA and ATX in a cohort of abstinent patients with lifetime AUD who were recruited from outpatient treatment programs. Given the high prevalence of comorbid liver diseases in the AUD, we explored their association with plasma concentrations of LPA and ATX. To this end, patients with AUD were divided into two subgroups based on the previous diagnosis of liver diseases.

## 2. Material and Methods

### 2.1. Participants and Recruitment

The present cross-sectional study included 136 Caucasian participants divided into two groups: 70 healthy control subjects (control group) and 66 patients diagnosed with AUD (alcohol group). The alcohol group was then divided into two subgroups: 43 patients with AUD but not liver disease (non-liver disease subgroup) and 23 patients with AUD and liver disease (liver disease subgroup).

Control subjects were recruited from two different sources, a multidisciplinary staff cohort of volunteers working at the Spanish National Public Health System (i.e., Hospital Regional Universitario de Málaga, Málaga, Spain; N = 15) and a second cohort obtained from volunteers donating data and plasma to the Red de Biobancos del Instituto de Salud Carlos III (i.e., Valdecilla biobank, Santander, Spain; N = 55). Patients with AUD were recruited from active outpatient treatment programs for alcohol at Hospital Universitario 12 de Octubre (Madrid, Spain). Control subjects were matched for age, body mass index (BMI), and sex with the alcohol group.

Participation was voluntary, but a non-randomized design was used to include patients with AUD and liver disease (i.e., fatty liver/steatosis, steatohepatitis, or cirrhosis). The patients with alcoholic liver disease were identified during the clinical assessment and included in the study after checking their personal health records. Patients with liver diseases were previously diagnosed and treated by the digestive health service at Hospital Universitario 12 de Octubre (Madrid, Spain).

All participants had to meet eligibility based on the following inclusion criteria: 18 years of age or older (up to 65 years) for all participants and diagnosis of lifetime AUD with a minimum of 4 weeks of abstinence for the alcohol group. The exclusion criteria included medical history of chronic inflammatory disorders ((e.g., cancer, coronary diseases and atherosclerosis, diabetes, arthritis, asthma, chronic obstructive pulmonary disease, neurodegenerative diseases, and diseases in the gastrointestinal tract and its accessory organs (except for liver)); infectious diseases (including COVID-19, HIV, hepatitis B, and hepatitis C); cognitive or language limitations precluding evaluation, pregnancy, or breastfeeding; and less than 4 weeks of abstinence from any drug, except for nicotine and caffeine for all participants. Specifically, the exclusion criteria for the control group also included the diagnosis of psychiatric disorders, the use of psychiatric medication in the last year, a medical history of liver diseases, and a personal history of problematic use of alcohol or other substances.

### 2.2. Ethics Statements

Written informed consents were obtained from each participant after a description of the study. All the participants had the opportunity to discuss any questions or issues. The study and protocols for recruitment were approved by the Ethics Committee of the Portal de Ética de la Investigación Biomédica de Andalucía-PEIBA (Consejería de Salud y Familias, Junta de Andalucía) in accordance with the “Ethical Principles for Medical Research Involving Human Subjects” adopted in the Declaration of Helsinki by the World Medical Association (Sixty-Fourth WMA General Assembly, Fortaleza, Brazil, October 2013) and the Regulation (EU) 2016/679 of the European Parliament and of the Council 27 April 2016 on the protection of natural persons with regard to the processing of personal data and on the free movement of such data, and repealing Directive 95/46/EC (General Data Protection Regulation). All data were given code numbers in order to maintain privacy and confidentiality.

### 2.3. Clinical Assessments

All participants were evaluated by trained and experienced psychologists using different psychiatric interviews based on the sample group. The Spanish version of the “Psychiatric Research Interview for Substance and Mental Diseases” (PRISM) was typically used for all participants. The PRISM is a semistructured interview based on the DSM-IV-TR criteria with good psychometric properties in the evaluation of substance use disorders and main psychiatric disorders in addicted population that demonstrated good to excellent validity and test–retest reliability [22,23]. While the alcohol group was specifically assessed with PRISM, control subjects were assessed with the Spanish version of the Composite International Diagnostic Interview (CIDI) for the detection of psychiatric disorders [24] and PRISM (module 1: *Overview* for sociodemographic and physiological variables).

### 2.4. Collection of Plasma Samples

Blood samples were obtained by experienced nurses in the morning after fasting for 8–12 h and before the clinical assessments. Venous blood samples were extracted into 10 mL K_2_ EDTA tubes (BD, Franklin Lakes, NJ, USA) and immediately centrifuged at 2200× *g* for 15 min (4 °C) to obtain plasma. Plasma samples were individually assayed to detect infectious diseases using commercial rapid tests for SARS-CoV-2 (Bio-Connect, The Netherlands), HIV, hepatitis B, and hepatitis C (Strasbourg, Cedex, France). Infected samples were discarded following laboratory safety protocols. Additionally, the blood alcohol concentration was measured according to the alcohol oxidase reaction using an Analox AM1 analyzer (Analox Instruments, Stourbridge, UK). The plasma samples were individually registered and stored in aliquots at −80 °C until determination of LPA and ATX.

### 2.5. Determination of LPA and ATX

Plasma concentrations of LPA and ATX were determined in duplicate using commercially available enzyme-linked immunosorbent assay (ELISA) kits following the manufacturer’s instructions.

Total LPA concentrations were measured using a Human LPA Elisa kit (CSB-EQ028005HU) from Cusabio Technology, Houston, USA. The human LPA Elisa kit indicated a sensitivity of 3.9 ng/mL and a detection range of 3.9–250.0 ng/mL without considering the dilution of samples (recommended 1:200). The intra-assay and inter-assay precisions showed a coefficient of variation (CV) <8% and <10%, respectively. The magnitude of the LPA concentrations in the plasma of healthy subjects was comparable to a previous study using the same human LPA Elisa kit [25].

ATX concentrations were determined using an Autotaxin Sandwich ELISA Kit (K-5600) from Echelon Biosciences, Utah, USA. The ATX Sandwich Elisa kit indicated a sensitivity of 1.56 ng/mL and a detection range of 1.56–100.0 ng/mL without considering the dilution of samples (recommended—1:50). The intra-assay and inter-assay precisions showed CV <4% and <8%, respectively.

The spectrophotometer used was a VersaMaxTunable Microplate Reader (Molecular Devices, LLC, San José, CA, USA), with a visible absorbance reading range between 340 and 850 nm. Raw data were obtained at 450 nm and analyzed using SoftMax Pro Software 5.4 (Molecular Devices, LLC, San José, CA, USA). In all cases, the samples were run in duplicate, and internal controls and a calibration curve were included in each ELISA kit. All samples showed an optical density (OD) higher than the limit of detection of both ELISA kits. Plasma concentrations of total LPA and ATX were expressed as ng/mL.

### 2.6. Biochemical Parameters Related to Liver Function

In addition to the determination of LPA and ATX, plasma samples of the patients with AUD were assessed for markers of liver function in a clinical analysis laboratory (Analysis Clinics Rodriguez Vergara S.L., Malaga, Spain). We examined the following biochemical parameters: aspartate transaminase (AST), gamma-glutamyltransferase (GGT), and alanine transaminase (ALT). The reference ranges from the laboratory were established as follows: 0–40, 0–45, 0–40 U/L, respectively.

### 2.7. Statistical Analysis

Data in Table 1 and Table 2 were expressed as the number and percentage of subjects (N (%)), mean and standard deviation (mean ± SD), or median and interquartile range (median (IQR)). The significance of differences in categorical and continuous variables was determined using the chi-square test and Student’s t-test (normal distribution) or Mann–Whitney U test (non-normal distribution), respectively.

Analyses of covariance (ANCOVA) were performed to evaluate the main effects and interaction of independent variables (group/subgroup factor) (i.e., control and alcohol; control, non-liver disease, and liver disease) on plasma concentrations of total LPA and ATX while controlling for age, BMI, and sex as covariates. Since LPA and ATX showed a positively skewed distribution, raw data were log_10_-transformed to approximate a normal distribution and to ensure statistical assumptions of the ANCOVA. Post hoc comparisons for multiple comparisons were performed using Sidak‘s correction test.

Correlation analyses between plasma concentrations of LPA and ATX (log_10_-transformed data) and relevant AUD-related variables (i.e., the last period of alcohol abstinence, the duration of problematic alcohol use, and the severity of AUD) were performed using the correlation coefficients of Pearson (*r*) and Spearman (rho) with continuous and categorical variables, respectively.

Receiver operating characteristics (ROC) analyses were performed to evaluate the discriminative power of binary logistic regression models through the area under the curve (AUC). In addition, the resulting probability data from these models were compared between groups/subgroups using Student’s t-test or Mann–Whitney U test.

The statistical analyses were performed with GraphPad Prism version 5.04 (GraphPad Software, San Diego, CA, USA) and IBM SPSS Statistics version 22 (IBM, Armonk, NY, USA). A *p*-value < 0.05 was considered statistically significant.

## 3. Results

### 3.1. Biological Characteristics and Plasma Concentrations of LPA and ATX

A biological description of the sample is shown in Table 1. A total of 136 participants were included according to the eligibility criteria and grouped into alcohol (N = 66) and control (N = 70) groups. In the alcohol group, the abstinent patients with AUD showed a mean age of 48 years, a mean BMI of 25 kg/m^2,^ and 85 percent were men. Because the control individuals were matched for age, BMI, and sex, there were no significant differences between groups. In contrast, total LPA and ATX concentrations were significantly different in both groups using non-parametric tests. Thus, the patients with AUD had significantly lower LPA (*p* < 0.01) and higher ATX (*p* < 0.01) concentrations than the control subjects.

**Figure 1:**
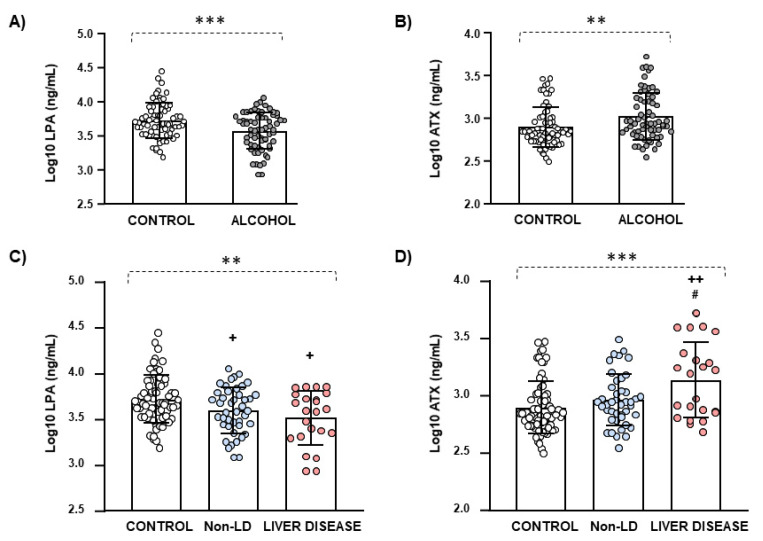
Plasma concentrations of LPA and ATX in the sample. (**A**) Log_10_-transformed concentrations of LPA in the control and alcohol groups; (**B**) Log_10_-transformed concentrations of ATX in the control and alcohol groups; (**C**) Log_10_-transformed concentrations of LPA in the control group, the non-liver disease (non-LD) subgroup, and the liver disease subgroup; and (**D**) Log_10_-transformed concentrations of ATX in the control group, the non-liver disease (non-LD) subgroup, and the liver disease subgroup. Lines and bars on the scatter plot are means and SD. Data were analyzed by ANCOVA while controlling for age, BMI, and sex as covariates. (***) *p* < 0.001 and (**) *p* < 0.01 denote significant main effects of the “group/subgroup” factor. (^+^) *p* < 0.05 and (^++^) *p* < 0.01 denote significant differences compared with the control group (post hoc test). (^#^) *p* < 0.05 denotes significant differences compared with the non-LD subgroup (post hoc test).

Raw data for plasma concentrations of LPA and ATX were log_10_-transformed to ensure statistical assumptions of the one-way ANCOVA while controlling for age, BMI, and sex. The analysis revealed a significant main effect of the group factor (control and alcohol group) on LPA (F_(1,131)_ = 10.677, *p* = 0.001) (Figure 1A) and ATX (F_(1,131)_ = 8.327, *p* = 0.005) (Figure 1B) concentrations, which confirmed the previous differences.

In addition, the association between LPA and ATX concentrations in the sample was explored using Pearson correlation analysis of log_10_-transformed data, but there were no significant correlations (data not shown).

### 3.2. Biological and Clinical Characteristics of the Alcohol Group Based on Liver Disease

Because the alcohol group displayed a high prevalence of liver diseases according to the non-randomized design of the present study, the abstinent patients with AUD were divided into two subgroups based on the diagnosis of liver diseases (i.e., fatty liver/steatosis (N = 13), alcoholic steatohepatitis (N = 8), and cirrhosis (N = 2)): liver disease (N = 23), and non-liver disease (N = 43) subgroups.

As shown in Table 2, both subgroups were clinically characterized with relevant variables associated with the diagnosis of lifetime AUD, psychiatric comorbidity, and liver damage.

Although there were no significant differences in age, BMI, and sex, more women were observed in the non-liver disease subgroup (21%). The comparison between the liver disease and non-liver disease subgroups revealed no significant differences in the clinical variables associated with the diagnosis of AUD and comorbid psychiatric disorders. Therefore, the patients with AUD showed a mean duration of problematic alcohol use of 18 years, a median of the last period of abstinence of 120 days, and a diagnosis of severe AUD based on DSM-IV-TR criteria for alcohol abuse and dependence. Regarding psychiatric comorbidity, 62 percent of the patients with AUD were diagnosed with comorbid mental disorders (mainly mood disorders (42%)), and 41 percent had additional substance use disorders (mainly cocaine (35%)). In contrast, there were significant differences in the concentrations of main biochemical markers of liver damage: AST (*p* < 0.001), GGT (*p* < 0.001), and ALT (*p* < 0.05) between both subgroups. Thus, patients with AUD and liver disease displayed abnormal values of AST (>40 U/L) and GGT (>45.0 U/L) and elevated values of ALT.

### 3.3. Plasma Concentrations of LPA and ATX Based on Liver Disease

The plasma concentrations of LPA and ATX were also examined in patients with AUD based on the diagnosis of liver diseases, but the comparison of raw data showed no significant differences between both subgroups (Table 2).

However, the one-way ANCOVA of the log_10_-transformed concentrations of LPA and ATX revealed significant main effects of the subgroup factor (liver disease, non-liver disease, and control). Thus, there was a significant main effect of the subgroup factor on the log_10_-transformed concentrations of LPA (F_(2,130)_ = 5.799, *p* = 0.004), and the post hoc test showed significantly lower LPA concentrations in the liver disease and non-liver disease subgroups than in the control group (*p* < 0.05) (Figure 1C). In addition, there was a significant main effect of the subgroup factor on the log_10_-transformed concentrations of ATX (F_(2,130)_ = 8.314, *p* < 0.001), and the post hoc test showed significantly higher ATX concentrations in the liver disease subgroup than in the non-liver disease subgroup (*p* < 0.05) and the control group (*p* < 0.01) (Figure 1D).

### 3.4. Correlation Analysis between AUD-Related Variables and Plasma Concentrations of LPA and ATX in the Alcohol Group Based on Liver Disease

As shown in Table 3, the association between AUD-related variables and plasma concentrations of LPA and ATX was explored in patients with AUD based on the diagnosis of liver diseases using Spearman and Pearson correlation analyses. Notably, we observed significant correlations in the non-liver disease subgroup. Thus, while the duration of alcohol abstinence was positively correlated with log_10_-transformed concentrations of LPA (r = +0.325, *p* < 0.05) and ATX (*r* = +0.325, *p* < 0.05), the severity of AUD (DSM criteria for AUD) was negatively correlated with log_10_-transformed concentrations of ATX (rho = −0.327, *p* < 0.05).

### 3.5. AUD-Related Variables and Plasma Concentrations of ATX and LPA as Predictors of Liver Disease

A first logistic regression model for the discrimination of patients with AUD from control subjects was constructed using LPA (log_10_-transformed concentrations), ATX (log_10_-transformed concentrations), age, BMI, and sex (Table S1). The comparison of the means of the resulting probability data showed significant differences (t_(134)_ = 4.995, *p* < 0.001) in the alcohol and control groups and the ROC analysis revealed a significant discriminative power for patients with AUD (AUC = 0.725 (95%CI = 0.640–0.811), *p* < 0.001) (Figure S1).

**Figure 2:**
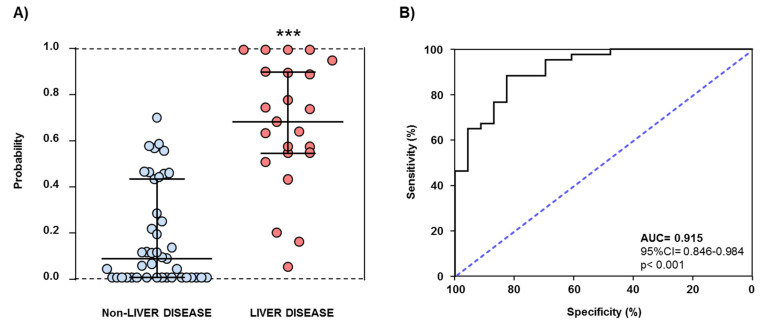
ROC analysis for a discriminative logistic model of patients with liver disease from patients without liver disease in the alcohol group. (**A**) Scatter plot of the predictive probabilities; and (**B**) ROC curve generated with probability values from a binary logistic model including LPA (log_10_-transformed), ATX (log_10_-transformed), problematic alcohol use, last period of alcohol abstinence (log_10_-transformed), and DSM criteria for AUD. Lines on the scatter plot are median and IQR. (***) *p* < 0.001 denotes significant differences using Mann–Whitney U test.

In a similar way, another logistic regression model for distinguishing patients with AUD and liver disease from patients with AUD but not liver disease was performed using LPA (log_10_-transformed concentrations), ATX (log_10_-transformed concentrations), the last period of alcohol abstinence, the duration of problematic alcohol use, and the DSM criteria for AUD (Table S2). The resulting probability data were significantly different when both alcohol subgroups were compared (U = 84, *p* < 0.001). In this case, the ROC analysis indicated an excellent discriminative power of the model (AUC = 0.915 (95% CI = 0.846–0.984), *p* < 0.001) and representative cutoff values showed high sensitivity and specificity (for example, 0.486 (83.3% sensitivity and 88.4% specificity) and 0.432 (87.0% sensitivity and 76.7% specificity)) (Figure 2).

## 4. Discussion

In agreement with recent preclinical and clinical evidence, the present study demonstrates that the LPA signaling is linked to AUD in humans. In this cross-sectional study, plasma concentrations of total LPA and ATX were measured in healthy control subjects and abstinent patients with AUD, who displayed an elevated prevalence of alcoholic liver diseases (35%: fatty liver/steatosis (56.5%), alcoholic steatohepatitis (34.8%), and cirrhosis (8.7%)) through a non-randomized strategy for the recruitment process. Therefore, we examined the association of LPA and ATX concentrations with AUD-related variables and psychiatric comorbidity in subgroups of patients with AUD based on the presence of liver diseases. Patients with liver diseases were previously diagnosed by the digestive health service, and we could confirm liver damage through markers of liver function (AST, GGT, and ALT). Since LPA signaling has been reported to be different according to age, weight, and sex [5,26], all statistical analyses were adjusted for these variables as cofactors.

The main findings of the study were as follows: (1) plasma concentrations of LPA and ATX were dysregulated in the alcohol group, and patients with AUD had significantly lower LPA and higher ATX concentrations than control subjects; (2) patients with AUD and comorbid liver disease had significantly higher ATX concentrations than patients with AUD but not liver disease; (3) there were significant correlations between relevant AUD-related variables, such as the duration of the last period of alcohol abstinence and the DSM criteria for AUD (severity), and the plasma concentrations of LPA and ATX in patients with AUD from the non-liver disease subgroup but not in the liver disease subgroup; and (4) a logistic regression model with LPA, ATX, and AUD-related variables showed an excellent discriminative power for distinguishing patients with AUD and comorbid liver disease, which suggests the ATX–LPA axis as a reliable predictor of alcoholic liver diseases.

The present data support a reduction in plasma concentrations of total LPA in patients with a history of AUD, which is in consonance with a recent clinical study from our group [5]. In contrast, plasma concentrations of ATX were higher in these patients than in controls. Although these results may seem contradictory because ATX is the major enzyme responsible for LPA extracellular, it is possible that high ATX levels converge with reduced LPA in pathological conditions where the ATX–LPA axis is strongly dysregulated [27]. LPA metabolism is determined by the dynamic balance of several factors and complicated mechanisms at both transcriptional and post-transcriptional levels that regulate the expression of LPA and ATX. Among the possible explanations for these differences, we can mention the functional activity of ATX and its inhibitory feedback control, the rapid degradation of LPA into monoacylglycerol by lipid phosphate phosphatase (LPP_1-3_), or the increased relevance of PLA isoforms involved in distinct synthesis routes of LPA with PA as substrate (e.g., group IIA secretory PLA_2_ (sPLA_2_-IIA) or membrane-bound PA-selective PLA_1_ (mPA-PLA_1_)) [28,29,30]. Furthermore, changes in the availability and diversity of substrates such as PA and lysophospholipids (i.e., lysophosphatidylcholine, lysophosphatidylserine, and lysophosphatidylethanolamine) for the synthesis enzymes of LPA should be considered in the context of AUD and other diseases. For example, PA is released by phospholipases D_1_ and D_2_ (PLD_1/2_) [10] from phospholipids, and the PLD signaling has been extensively studied in many pathological conditions such as cancer, inflammatory diseases, neurodegenerative disorders [31,32,33], and (neuro)toxicity. Interestingly, PLD generates phosphatidyl ethanol in the presence of ethanol at the expense of PA, and this metabolite is used as a marker for alcohol use and abuse [34]. Therefore, a persistent decrease in the PA accumulation because of a chronic alcohol intake could contribute to a low expression of LPA in patients with AUD in early abstinence, but further investigation is necessary.

Previous studies from our group have reported that the presence of common accessory digestive organ diseases in patients with AUD correlates with alterations in the immune system through the dysregulated expression of inflammatory and immunomodulatory factors in plasma. Indeed, we have observed that alcohol-induced liver and pancreas diseases are associated with elevated concentrations of interleukin (IL)-8 [35] and IL-6 [36], and decreased concentrations of IL-17A [36] and growth factor brain-derived neurotrophic factor (BDNF) [2], which suggests a potential role of these molecules as indicators or biomarkers of these comorbid diseases in accessory gastrointestinal organs. In the present study, patients with AUD had a high incidence of liver diseases (35%), and the diagnosis of fatty liver/steatosis, alcoholic steatohepatitis, or cirrhosis was associated with higher plasma concentrations of ATX but not differences in LPA. In line with these results, increased levels of ATX in the blood have been described as a key feature of liver diseases and predict fibrosis in NAFLD [21], likely because of impaired ATX clearance by the hepatic cells [37].

Alcohol consumption and some drinking habits, such as the duration and the amount of alcohol consumption, are considered as primary factors responsible for the development of alcohol-induced liver damage [38,39]. Because the presence of comorbid liver disease in patients with AUD was associated with differences in plasma concentrations of ATX, we examined the correlation between the ATX–LPA axis and relevant alcohol-related variables (i.e., the last period of alcohol abstinence, the problematic alcohol use, and the severity of AUD) in the subgroups of patients with AUD based on the diagnosis of liver disease. Interestingly, while no significant associations were found in the liver disease subgroup, there were significant correlations between plasma LPA and ATX concentrations and some alcohol-related variables. Specifically, there was a positive correlation between the duration of alcohol abstinence and the plasma concentrations of LPA and ATX and an inverse correlation between the severity of AUD and the plasma concentrations of ATX, which was also observed in the total alcohol group. Therefore, in the absence of liver diseases, lower LPA concentrations were associated with recent alcohol abstinence, and lower ATX concentrations were associated with high severity of AUD. The nature of these significant correlations is unknown because most studies on LPA signaling in alcohol are only focused on the development and progression of liver diseases without considering characteristics of chronic alcohol use or AUD.

In accordance with these results, a previous study in the same cohort of patients with AUD reported a decrease in LPA concentrations compared with healthy control subjects. However, this other study was conducted in patients without accessory digestive organ diseases and showed that lower concentrations of LPA predict impaired cognitive function and correlate directly with BDNF concentrations [5]. Therefore, while alcohol-related cognitive impairment is associated with a decrease in plasma concentrations of LPA signaling, our data suggest that protracted alcohol abstinence may restore both LPA concentrations and cognitive functioning. Consistent with this hypothesis during the abstinence period, increased levels of LPA have been linked to promoting neuroplasticity and desirable behavioral outcomes related to cognition and emotion [15]. Interestingly, severe alcoholic and nonalcoholic liver diseases have been linked to a higher risk of cognitive impairment and hepatic encephalopathies through inflammatory responses, cerebrovascular alterations, and insulin resistance, among other mechanisms [40,41]. In this study, the presence of liver diseases abolished all the correlations between the LPA signaling and alcohol-related variables, such as the duration of alcohol abstinence and the dysfunction of the LPA signaling observed through high concentrations of ATX. Therefore, although our findings present ATX as a reliable characteristic of liver disease in patients with AUD, the cognitive function will need to be assessed to confirm the association between the ATX–LPA axis and cognition in AUD based on the presence of liver diseases.

Given the differences in the expression of this lipid signaling system, logistic regression models were constructed in order to explore the discriminative power of the ATX–LPA axis in the context of AUD and comorbid alcoholic liver diseases. Thus, although the ROC analysis of a regression model showed a good discriminative power to discriminate between controls and patients with AUD, a second regression model with LPA, ATX, and AUD-related variables (i.e., abstinence, problematic alcohol use, and AUD severity) showed an excellent discriminatory power to distinguish patients with alcoholic liver disease from patients without alcoholic liver disease.

In conclusion, this study strengthens the importance of the ATX–LPA axis as a reliable biomarker for diagnosis and/or prediction of AUD and its associated complications, such as alcoholic liver diseases, in patients who seek treatment. In addition to these clinical findings, because previous preclinical studies have reported that ethanol administration dysregulates blood LPA and ATX levels [7,42] and the pharmacological inhibition of LPA1-mediated signaling modulates vulnerability to alcohol [6,7], the characterization of the LPA signaling is worthy of further investigation as a therapeutic target for patients with AUD; for example, developing drugs for the ATX–LPA axis.

### Limitations and Future Directions

We are aware of the limitations of the present study and the necessity of further investigation. First, patients with AUD from our cohort are mainly middle-aged men, which is a realistic reflection of individuals seeking treatment for problematic alcohol use; however, our findings cannot be extrapolated to women and other intervals of age because a larger sample size and better characterization are required. Second, the high prevalence of psychiatric comorbidity could influence our data, although this is a characteristic of addictive disorders, and no differences were found between patients with AUD based on the presence of liver disease. Third, a better clinical characterization of the alcoholic liver disease in a larger sample (subtypes and stages (i.e., fatty liver/steatosis, alcoholic steatohepatitis, and cirrhosis), pharmacological treatment, diet, and nutritional support, risk factors, etc.) is also required to identify the specific role of the ATX–LPA axis and its association with these clinical variables. Fourth, because the LPA metabolism involves a dynamic balance of several enzymes (e.g., PLD_1/2_, ATX, mPA-PLA_1_, sPLA_2_-IIA, and LPP_1-3_,) and metabolites (e.g., PA, lysophospholipids, and monoacylglycerols), a complete molecular characterization is necessary to establish the mechanism of regulation of LPA signaling; in addition, the determination of distinct LPA species such as LPA 18:2 and 20:4 could extend this molecular characterization. Fifth, cross-validation of the proposed discriminative models is necessary to ensure reliability and stability of these data; however, it is important to note at this point that the magnitude of LPA concentrations reported among studies in humans is different and could be explained by several factors (e.g., protocols for the preparation of samples (type of blood collection tube and anticoagulant, centrifugation conditions) and methods for the quantification (liquid chromatography with mass spectrometry—LC/MS, capillary electrophoresis, competitive enzyme-linked immunosorbent assay—ELISA)). Finally, following these exploratory cross-sectional studies, we consider that a longitudinal study is essential in future investigations to observe how plasma concentrations of LPA and ATX may change over time.

## Figures and Tables

**Table 1:** Biological characteristics and plasma concentrations of LPA and ATX.

Variable		Group		*p*-Value
		Control (N = 70)	Alcohol (N = 66)	
Age (years)	Mean ± SD	47.8 ± 6.5	48.1 ± 5.7	0.760 ^a^
BMI (kg/m^2^)	Mean ± SD	24.2 ± 2.1	25.1 ± 3.4	0.060 ^a^
Sex (men)	N (%)	60 (85.7)	56 (84.8)	0.887 ^b^
LPA (ng/mL)	Median (IQR)	4917.1 (4393.0)	3972.7 (3726.4)	0.009 ^c^
ATX (ng/mL)	Median (IQR)	696.5 (472.6)	885.2 (1049.8)	0.004 ^c^

(^a^) *p*-value from Student t-test. (^b^) *p*-value from chi-square test. (^c^) *p*-value from Mann–Whitney U test. Abbreviations: ATX = autotaxin; BMI = body mass index; IQR = interquartile range; LPA = total lysophosphatidic acid; SD = standard deviation.

**Table 2:** Clinical and biological characteristics of the alcohol subgroups.

Variable		Subgroup		*p*-Value
		Non-Liver Disease (N = 43)	Liver Disease (N = 23)	
Age (years)	Mean ± SD	48.1 ± 6.4	48.2 ± 4.2	0.911 ^a^
BMI (kg/m^2^)	Mean ± SD	24.6 ± 3.3	25.9 ± 3.5	0.155 ^a^
Sex (men)	N (%)	34 (79.1)	22 (95.7)	0.073 ^b^
Problematic alcohol use (years)	Mean ± SD	17.4 ± 11.2	19.3 ± 9.9	0.510 ^a^
Last period of alcohol abstinence (days)	Median (IQR)	120.0 (120.0)	120.0 (120.0)	0.760 ^c^
DSM criteria for AUD (0–11)	Median (IQR)	8.0 (2.0)	7.0 (3.0)	0.313 ^c^
Mental disorders	N (%)	28 (65.1)	13 (56.5)	0.493 ^b^
Mood disorders	N (%)	20 (46.5)	8 (34.8)	0.713 ^b^
Anxiety disorders	N (%)	11 (25.6)	4 (17.4)	0.728 ^b^
Psychotic disorders	N (%)	6 (14.0)	1 (4.3)	0.425 ^b^
Personality disorders	N (%)	8 (18.6)	5 (21.7)	0.760 ^b^
Substance use disorders	N (%)	17 (39.5)	10 (43.5)	0.756 ^b^
Cocaine	N (%)	15 (34.9)	8 (34.8)	0.993 ^b^
Cannabis	N (%)	6 (14.0)	5 (21.7)	0.419 ^b^
AST (0.0–40.0 U/L)	Mean ± SD	28.4 ± 2.6	44.1 ± 5.8	<0.001 ^a^
GGT (0.0–45.0 U/L)	Mean ± SD	36.2 ± 5.1	49.1 ± 6.5	<0.001 ^a^
ALT (0.0–40.0 U/L)	Mean ± SD	34.9 ± 3.0	38.4 ± 6.2	0.017 ^a^
LPA (ng/mL)	Median (IQR)	3941.1 (3554.1)	4004.4 (3994.0)	0.364 ^c^
ATX (ng/mL)	Median (IQR)	883.2 (534.0)	1574.5 (1982.2)	0.079 ^c^

(^a^) *p*-value from Student t-test. (^b^) *p*-value from chi-square test. (^c^) *p*-value from Mann–Whitney U test. Abbreviations: ALT = alanine transaminase; AST = aspartate transaminase; ATX = autotaxin; AUD = alcohol use disorder; BMI = body mass index; DSM = Diagnostic and Statistical Manual of Mental Disorders; GGT = gamma-glutamyltransferase; IQR = interquartile range; LPA = total lysophosphatidic acid; SD = standard deviation.

**Table 3:** Correlation analysis between AUD-related variables and plasma concentrations of LPA and ATX.

Variable	LPA (ng/mL) ^a^			ATX (ng/mL) ^a^		
	Alcohol Group	Alcohol Subgroup		Alcohol Group	Alcohol Subgroup	
		Non-Liver Disease	Liver Disease		Non-Liver Disease	Liver Disease
Problematic alcohol use (years)	*r* = +0.082 *p* = 0.514	*r* = +0.158 *p* = 0.312	*r* = −0.017 *p* = 0.938	*r* = −0.049 *p* = 0.696	*r* = −0.185 *p* = 0.235	*r* = +0.072 *p* = 0.745
Last period of alcohol abstinence (days) ^a^	*r* = +0.157 *p* = 0.207	*r* = +0.325 *p* = 0.033	*r* = −0.183 *p* = 0.403	*r* = +0.140 *p* = 0.263	*r* = +0.326 *p* = 0.033	*r* = −0.158 *p* = 0.470
DSM criteria for AUD (0–11)	rho = +0.067 *p* = 0.590	rho = +0.097 *p* = 0.538	rho = −0.089 *p* = 0.686	rho = −0.264 *p* = 0.032	rho = −0.327 *p* = 0.033	rho = −0.212 *p* = 0.332

(^a^) Log_10_ values. Abbreviations: ATX = autotaxin; DSM = Diagnostic and Statistical Manual of Mental Disorders; LPA = total lysophosphatidic acid; r = Pearson’s correlation coefficient; rho = Spearman’s correlation coefficient.

## Supplementary Materials

The following are available online at https://www.mdpi.com/article/10.3390/biomedicines9091207/s1, Figure S1: ROC analysis for a discriminative logistic model of patients with AUD from control subjects in the sample, Table S1: Logistic regression model for distinguishing patients with AUD, Table S2: Logistic regression model for distinguishing patients with AUD and liver disease.
