# Supplementary material for: Plasma Concentrations of Lysophosphatidic Acid and Autotaxin in Abstinent Patients with Alcohol Use Disorder and Comorbid Liver Disease
Source: Biomedicines. 2021 Sep 13;9(9):1207. doi: 10.3390/biomedicines9091207 (PMC8469650; doi:10.3390/biomedicines9091207)
Supplement: Supplementary file 1 [file biomedicines-09-01207-s001.zip › figure legends (2).pdf]

FIGURE LEGENDS

Figure 1. Plasma concentrations of LPA and ATX in the sample. **(A)** Log_{10}-transformed concentrations of LPA in the Control and Alcohol groups; **(B)** Log_{10}-transformed concentrations of ATX in the Control and Alcohol groups; **(C)** Log_{10}-transformed concentrations of LPA in the Control group, the non-Liver Disease (non-LD) subgroup and the Liver Disease subgroup; and **(D)** Log_{10}-transformed concentrations of ATX in the Control group, the non-Liver Disease (non-LD) subgroup and the Liver Disease subgroup. Lines and bars on the scatter plot are means and SD. Data were analyzed by ANCOVA while controlling for age, BMI and sex as covariates. (***) $p<0.001$ and (**) $p<0.01$ denote significant main effects of the "group/subgroup" factor. ($^{+}$) $p<0.05$ and ($^{++}$) $p<0.01$ denote significant differences compared with the Control group (*post hoc* test). ($^{\#}$) $p<0.05$ denotes significant differences compared with the non-LD subgroup (*post hoc* test).

Figure 2. ROC analysis for a discriminative logistic model of patients with Liver Disease from patients without Liver Disease in the Alcohol group. **(A)** Scatter plot of the predictive probabilities; and **(B)** ROC curve generated with probability values from a binary logistic model including LPA (log_{10}-transformed), ATX (log_{10}-transformed), problematic alcohol use, last period of alcohol abstinence (log_{10}-transformed) and DSM criteria for AUD. Lines on the scatter plot are median and IQR. (***) $p<0.001$ denotes significant differences using Mann-Whitney U test.

Figure S1. ROC analysis for a discriminative logistic model of patients with AUD from control subjects in the sample. Scatter plot of the predictive probabilities and ROC curve generated with probability values from a binary logistic model including LPA (log_{10}), ATX (log_{10}), age, BMI and sex. Lines on the scatter plot are mean and SD. (***) $p<0.001$ denotes significant differences using Student's t test.
