# Supplementary material for: Plasma Concentrations of Lysophosphatidic Acid and Autotaxin in Abstinent Patients with Alcohol Use Disorder and Comorbid Liver Disease
Source: Biomedicines. 2021 Sep 13;9(9):1207. doi: 10.3390/biomedicines9091207 (PMC8469650; doi:10.3390/biomedicines9091207)
Supplement: Supplementary file 1 [file biomedicines-09-01207-s001.zip › Table S1 (2).pdf]

Table S1. Logistic regression model for distinguishing patients with AUD									
	VARIABLE	B	SE	W	df	p-value	Exp(B)	95% CI for Exp(B)	
								Lower	Upper
BINARY LOGISTIC REGRESSION MODEL	LPA [a]	2.418	0.783	9.525	1	0.002	11.221	2.417	52.105
	ATX [a]	-2.233	0.797	7.862	1	0.005	0.107	0.022	0.511
	Age	-0.006	0.031	0.038	1	0.845	0.994	0.935	1.057
	BMI	-0.133	0.070	3.600	1	0.058	0.875	0.763	1.004
	Sex	0.315	0.521	0.366	1	0.545	1.370	0.494	3.803
	Constant	1.123	1.778	0.075	1	0.784	3.074	-	-

([a]) Log_{10} values

Abbreviations: ATX=Autotaxin; B=Coefficient; BMI=Body mass index; CI=Confidence interval; df=Degrees of freedom; LPA=Total lysophosphatidic acid; SE=Standard error; W=Wald test
