# Supplementary material for: Plasma Concentrations of Lysophosphatidic Acid and Autotaxin in Abstinent Patients with Alcohol Use Disorder and Comorbid Liver Disease
Source: Biomedicines. 2021 Sep 13;9(9):1207. doi: 10.3390/biomedicines9091207 (PMC8469650; doi:10.3390/biomedicines9091207)
Supplement: Supplementary file 1 [file biomedicines-09-01207-s001.zip › Table S2 (2).pdf]

Table S2. Logistic regression model for distinguishing patients with AUD and liver disease

	VARIABLE	B	SE	W	df	p-value	Exp(B)	95% CI for Exp(B)	
								Lower	Upper
BINARY LOGISTIC REGRESSION MODEL	**LPA [a]**	-2.540	1.620	2.459	1	0.117	0.079	0.003	1.887
	**ATX [a]**	2.178	1.659	1.724	1	0.189	8.832	0.342	228.242
	**Problematic alcohol use**	0.045	0.041	1.193	1	0.275	1.046	0.965	1.134
	**Last period of alcohol abstinence**	-0.938	0.874	1.152	1	0.283	0.391	0.071	2.171
	**DSM criteria for AUD [b]**			6.046	9	0.735			
	DSM criteria for AUD (1)	41.941	42679	0.000	1	0.999	1.64E+18	-	-
	DSM criteria for AUD (2)	-1.083	42679	0.000	1	1.000	0.339	-	-
	DSM criteria for AUD (3)	42.131	26138	0.000	1	0.999	1.98E+18	-	-
	DSM criteria for AUD (4)	19.899	14353	0.000	1	0.999	438561996	-	-
	DSM criteria for AUD (5)	22.320	14353	0.000	1	0.999	4937405080	-	-
	DSM criteria for AUD (6)	19.413	14353	0.000	1	0.999	269704268	-	-
	DSM criteria for AUD (7)	-0.292	19346	0.000	1	1.000	0.746	-	-
	DSM criteria for AUD (8)	21.623	14353	0.000	1	0.999	2460003041	-	-
	DSM criteria for AUD (9)	21.666	14353	0.000	1	0.999	2566477713	-	-
	**Constant**	-17.610	14353	0.000	1	0.999	0.000	-	-

([a]) Log_{10} values
([b]) Categorical variable

Abbreviations: ATX=Autotaxin; AUD=Alcohol use disorder; B=Coefficient; CI=Confidence interval; df=Degrees of freedom; DSM=Diagnostic and Statistical Manual of Mental Disorders; LPA=Total lysophosphatidic acid; SE=Standard error; W=Wald test
