# Supplementary figures and images for: Plasma Concentrations of Lysophosphatidic Acid and Autotaxin in Abstinent Patients with Alcohol Use Disorder and Comorbid Liver Disease
Source: Biomedicines. 2021 Sep 13;9(9):1207. doi: 10.3390/biomedicines9091207 (PMC8469650; doi:10.3390/biomedicines9091207)

## Slide 1
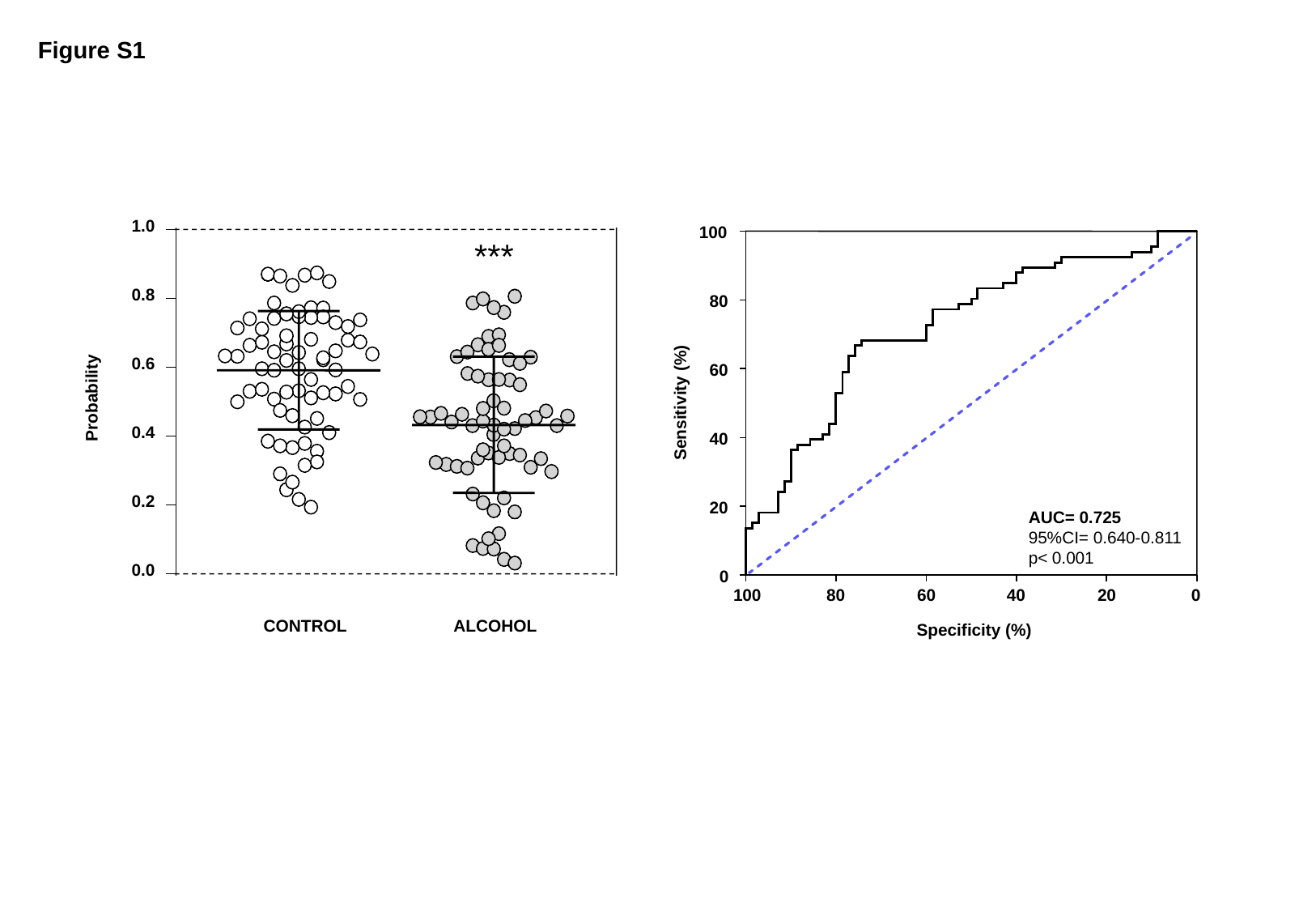

Figure S1
1.0
***
0.8
0.6
Probability
0.4
0.2
0.0
CONTROL
ALCOHOL
100
80
60
Sensitivity (%)
40
20
AUC= 0.725
95%CI= 0.640-0.811
p< 0.001
0
100
80
60
40
20
0
Specificity (%)

Supplement: Supplementary file 1 [file biomedicines-09-01207-s001.zip › Figure S1 (2).pptx]
